# SlBL4 is involved in leaf polarity development in tomato

**DOI:** 10.3389/fpls.2026.1765515

**Published:** 2026-04-28

**Authors:** Nan Hu, Xin Gao, Kexin Chang, Ruonan Yu, Lele Li, Jiazhi Li, Zhengguo Li, Dongyang Dai, Hada Wuriyanghan, Fang Yan

**Affiliations:** 1 College of Horticulture and Forestry, Tarim University, Alar, China; 2 Key Laboratory of Forage and Endemic Crop Biotechnology, Ministry of Education, School of Life Sciences, Inner Mongolia University, Hohhot, China; 3 Key Laboratory of Plant Hormones and Molecular Breeding of Chongqing, School of Life Sciences, Chongqing University, Chongqing, China

**Keywords:** *SlBL4*, leaf polarity, tomato, RNAi, IAA

## Abstract

The adaxial–abaxial axes play a crucial role in normal leaf growth. While recent studies have explored the molecular mechanisms underlying these developmental processes, the functions of *BELL* homologous genes remain insufficiently understood. In this study, we demonstrate that silencing the *SlBL4* gene in tomatoes (*Solanum lycopersicum* cv. Micro-Tom) affects leaf polarity development. RNA sequencing (RNA-seq) analysis indicates that SlBL4 regulates numerous differentially expressed genes (DEGs) associated with leaf polarity, auxin transporters, LOB domain-containing proteins, chlorophyll accumulation, and chloroplast development. Furthermore, SlBL4 positively regulated the expression of *SlGH3.1*, *SlGH3.2*, and *SlLOB4* genes associated with leaf growth polarity. These findings elucidate the roles of SlBL4 and propose a novel function for *BELL* genes in regulating plant leaf polarity.

## Introduction

Leaf formation and growth are dynamic processes that begin at the shoot apical meristem (SAM) and develop along three polarity axes, which are crucial for plant growth (Hudson, 1999; Bowman and Floyd, 2008). A transcriptional regulatory network of genes controlling leaf polarity across the three axes has been identified (Fritz et al., 2018; Ali et al., 2020). The precise specification of the adaxial and abaxial polarity axes plays a vital role in the development and functionality of leaves. Certain transcription factors (TFs) and small RNAs are essential for the development of adaxial–abaxial polarity (Moon and Hake, 2011). The *asymmetric leaves1* (*as1*)/*PHANTASTICA* (*PHAN*) and *as2* mutants exhibit abnormal axis formation in *Arabidopsis thaliana*. In the adaxial domain, the AS1–AS2 protein complex negatively regulates *ARFs* and *miR166A* (Iwasaki et al., 2013; Husbands et al., 2015). *KANADI* encodes a nuclear-localized GARP TF regulating the development of leaf polarity (Pekker et al., 2005; Iwakawa et al., 2007). *YABBY2* and *YABBY3* determine the development of abaxial cells of leaves (Siegfried et al., 1999). Furthermore, *AS2* negatively regulates *KANAD2* and *YABBY5* in establishing leaf polarity (Pekker et al., 2005; Wu et al., 2008). REVOLUTA (REV), ROLLED1 (RLD1), PHABULOSA (PHB), and PHAVOLUTA (PHV), which belong to the HD-ZIP III family, can affect leaf adaxial–abaxial polarity development (McConnell et al., 2001; Otsuga et al., 2001). Additionally, the transport of auxin between leaf primordia and the SAM results in a transient low auxin domain on the adaxial side, contributing to the adaxial–abaxial patterning of leaf polarity. Leaf polarity can be disrupted by ectopic adaxial activation in *Arabidopsis* or exogenous auxin treatment in tomato (Qi et al., 2014). *PIN1* (*PIN-FORMED*, *PIN*), an auxin transporter, indicates involvement in cell polarity due to its polar distribution (Xiong and Jiao, 2019; Wenzel et al., 2024). The influence of ad/abaxial specific genes on leaf polarity remains to be fully explored.

Recent studies have extensively explored *BEL1*-*like* (*BELL*) TFs across various plant species for their roles in the initiation, maintenance, and development of the SAM, flowering initiation, inflorescence architecture, chloroplast development, chlorophyll accumulation, and abscission-zone development (Ung et al., 2011; Khan et al., 2012; Yoon et al., 2015; Meng et al., 2018; Ikeda et al., 2019; Yan et al., 2020, Yan et al., 2021; Niu et al., 2022, Niu et al., 2022; Yu et al., 2024). In *Arabidopsis*, *BLH2* and *BLH4* establish leaf shape by repressing *BREVIPEDICELLUS* (*BP*) expression (Kumar et al., 2007). In the KNOX mutant *bp*, increased expression of *KNAT2* and *KNAT6* results in a downward-pointing inflorescence architecture (Ragni et al., 2008). ARABIDOPSIS THALIANA HOMEOBOX (ATH1), a BELL-type protein, interacts with KNAT2 to regulate inflorescence orientation (Li et al., 2012). PENNYWISE (PNY) and POUND-FOOLISH (PNF) contribute to forming the correct phyllotaxis in *Arabidopsis* (Smith and Hake, 2003). A *pinnate1* mutant, a BLH family member, alters leaf palm shape in *Medicago truncatula* (He et al., 2020). SlBLH3 inhibits leaflet growth via its interaction with SlKN2 (Kimura et al., 2008; Ezura et al., 2022). However, the role of BELL proteins in leaf polarity development in tomato remains largely unexplored.

*SlBL4*, a member of the *BELL*, plays diverse roles in chloroplast development, chlorophyll accumulation, cell wall degradation, fruit pedicel organogenesis, and abscission (Nakano et al., 2013; Meng et al., 2018; Yan et al., 2020; Yan et al., 2021). This study identifies a new regulator of leaf polarity. Tomato plants with downregulated *SlBL4* expression exhibit up-curled leaves and altered expression of auxin transporters, leaf polarity, and LOB domain-containing genes. SlBL4 directly regulates the expression of *SlGH3.1*, *SlGH3.2*, and *SlLOD4* genes related to polarity during leaf development.

## Results

### Expression pattern of *SlBL4* in tomato

**Figure 1:**
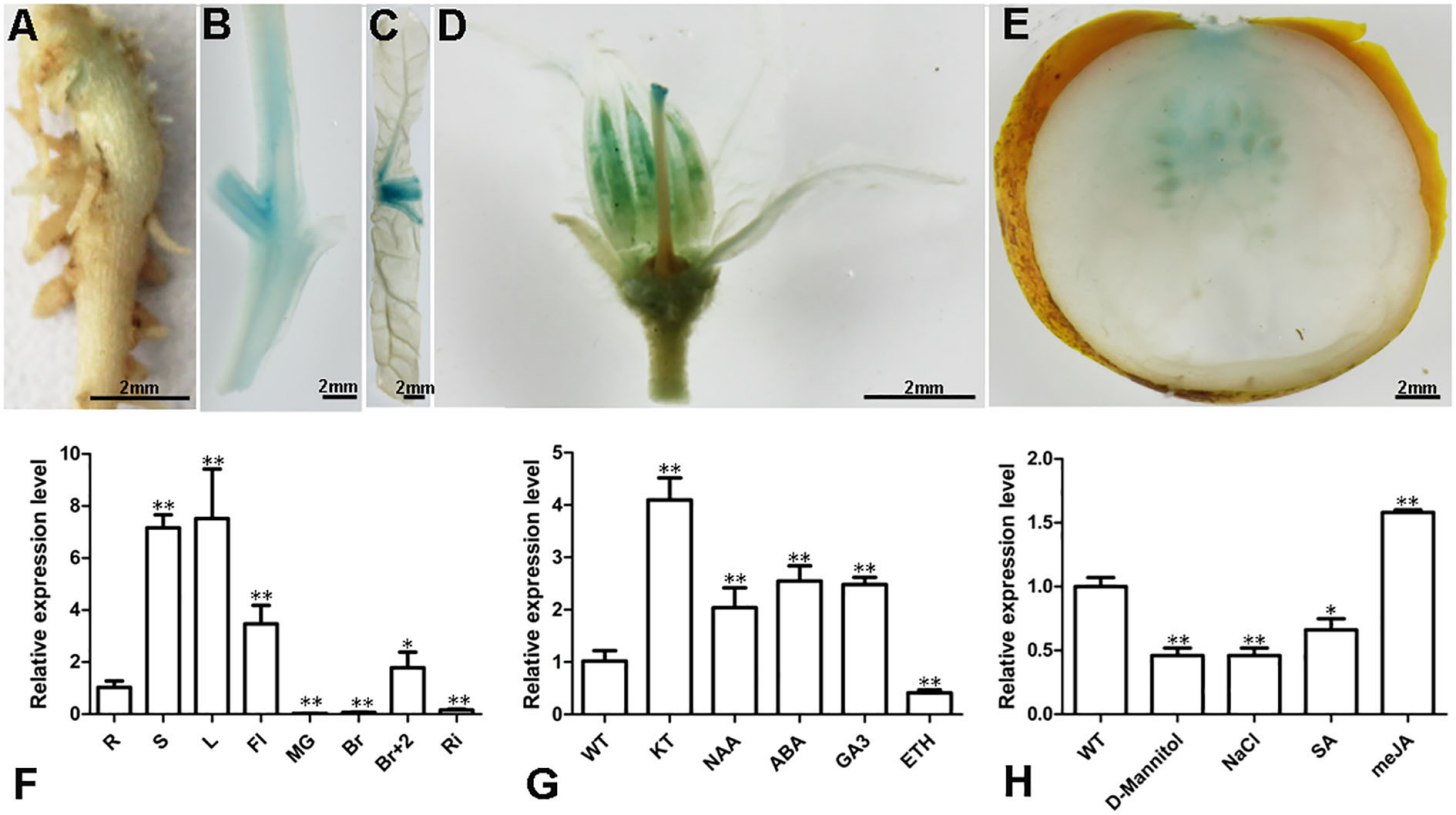
Analysis of *SlBL4* expression. **(A–E)** *SlBL4*pro::*GUS* expression. Scale bars: 2 mm. **(A)** Root; **(B)** stem; **(C)** leaf; **(D)** flower; **(E)** fruit; **(F)** qPCR analysis of the *SlBL4* in different tissues of tomato; R, root; S, stem; L, leaf; Fl, flower; MG, mature green fruit; Br, breaker fruit; Br+7, breaker fruit 7 days. **(G)** qPCR detection of the *SlBL4* in response to plant hormone treatments. WT, wild type; KT, kinetin; NAA, 1-Naphthaleneacetic acid; ABA, abscisic acid; GA_3_, Gibberellin acid 3; ETH, ethephon; 0.1 mM solutions of KT, NAA, ABA, GA_3_, ethylene, D-Mannitol, NaCl, SA, and meJA individually in treatments. **(H)** qPCR analysis of the *SlBL4* under stress treatments. SA, salicylic acid; meJA, methyl jasmonate. The quantitative PCR data represent mean values for three independent biological replicates. Asterisks indicate statistical significance at *p* < 0.01 (**) or 0.05 > *p* > 0.01 (*) as determined by *t* test.

To investigate the expression of *SlBL4*, we analyzed the expression pattern of an *SlBL4*-*GUS* transcriptional fusion, where the *SlBL4* promoter is linked to the *GUS* gene encoding β-glucuronidase. The expression of *SlBL4*-*GUS* was higher in stems, leaves, and flowers compared to roots and fruits (**Figures 1A–E**). Quantitative real-time PCR (qPCR) was performed to assess transcription levels in various tomato tissues. Expression levels were elevated in stems (S), leaves (L), flowers (Fl), and relatively lower in roots (R) and fruits (**Figure 1F**). The qPCR and GUS assay results were consistent. The mRNA level of *SlBL4* is upregulated by KT, NAA, ABA, GA3, and JA, and downregulated by SA, ethylene, NaCl, and mannitol treatments (**Figures 1G, H**).

### Downregulation of *SlBL4* causes curly leaves in tomato

**Figure 2:**
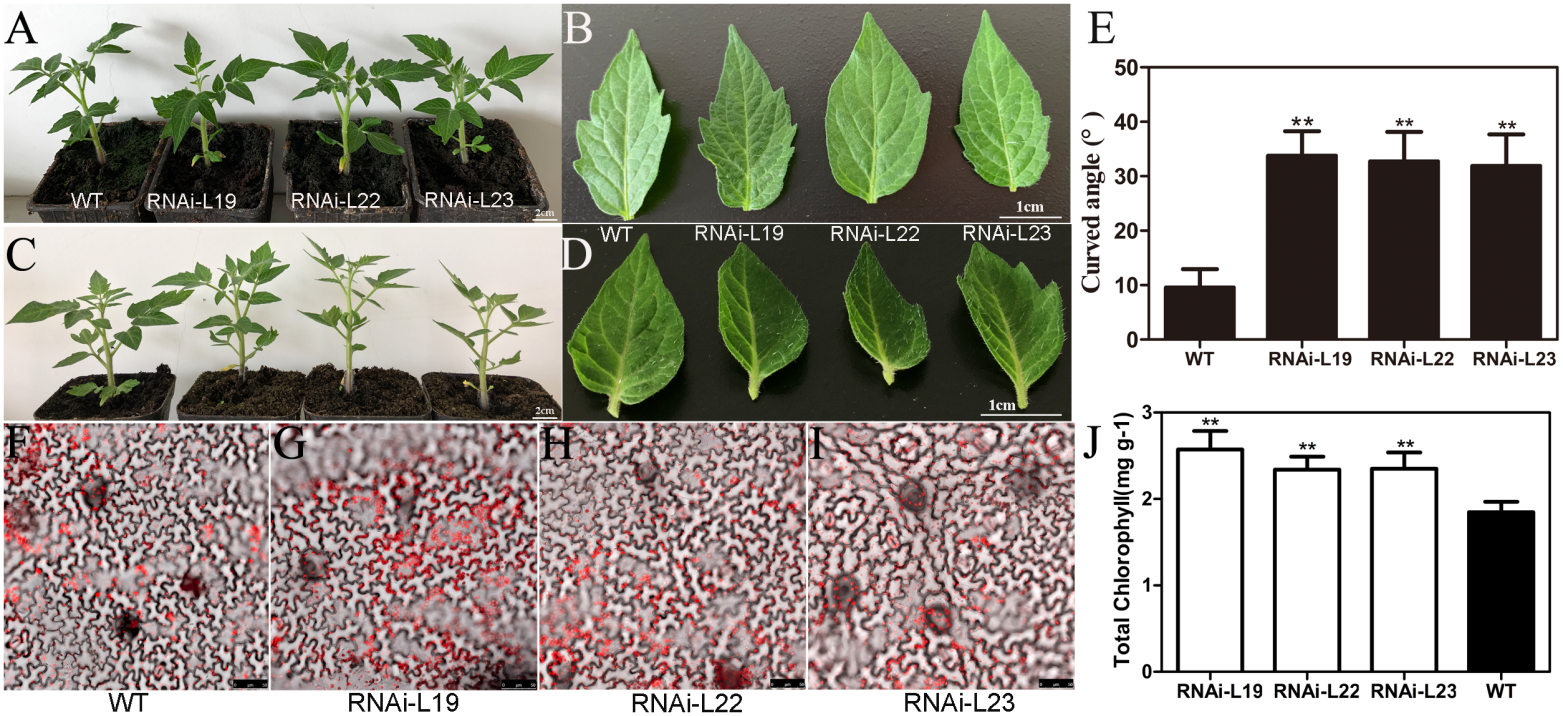
The phenotype of the leaf in transgenic plants. **(A–D)** The phenotypes of curled-up leaves; scale bars: 1 cm. **(E)** The bending angle of the leaf; **(F–I)** confocal laser-scanning microscope images of the leaf of the WT and three RNAi lines, showing chlorophyll autofluorescence. The scale bars are 50 μm. **(J)** Chlorophyll content in the leaf at 35 days. All data are means (± SE), *n* = 3. Significant differences compared with the WT were determined using Student’s *t* test: **p < 0.01.

**Figure 3:**
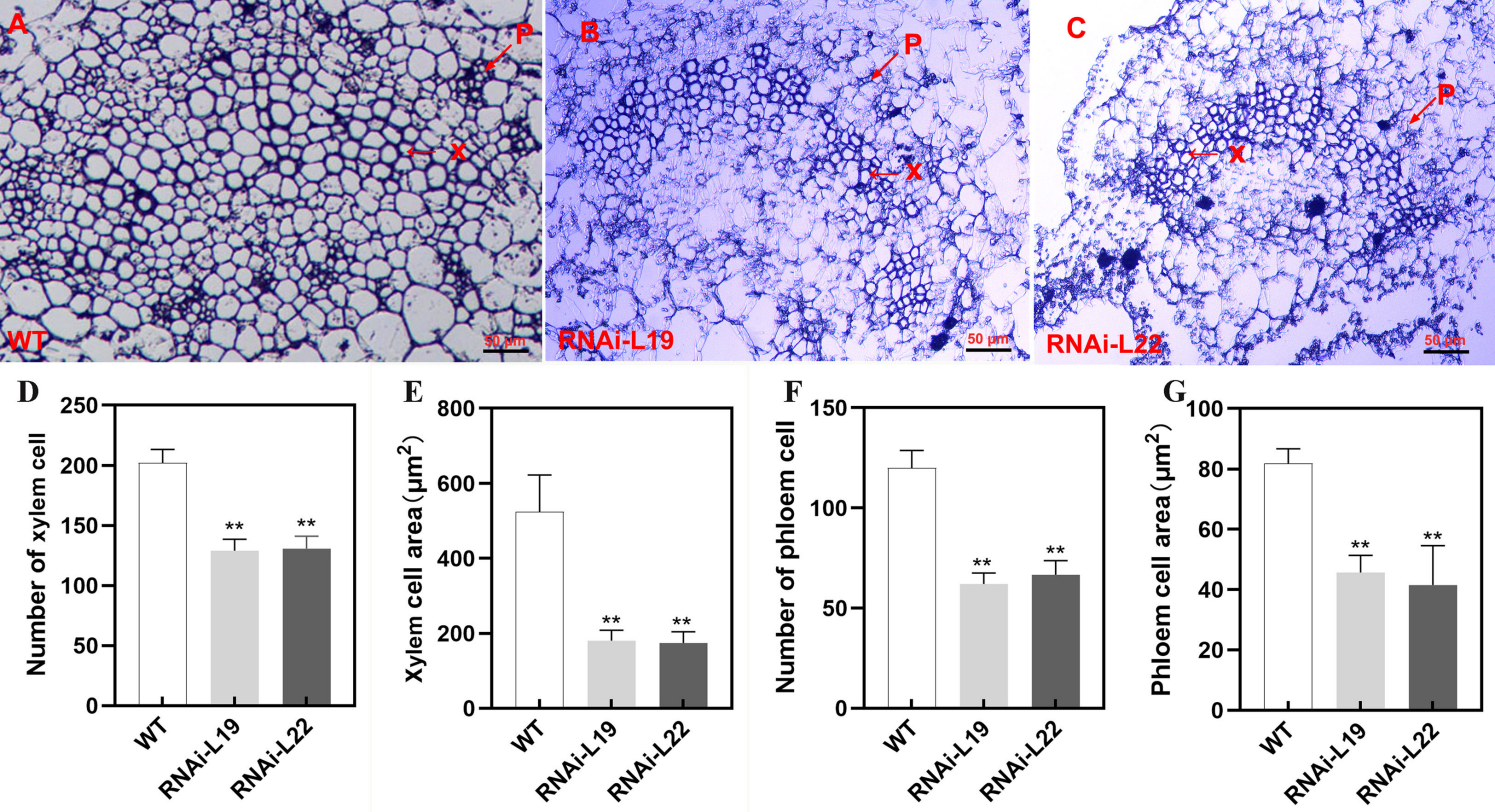
Anatomic analysis of *SlBL4* RNAi transgenic plants leaf. **(A)** Micro-section of a leaf in the wild type. **(B)** Micro-section of leaf in *SlBL4* RNAi-L19. **(C)** Micro-section of leaf in *SlBL4* RNAi-L22; scale bars: 50 μm. **(D)** Number of xylem cells. **(E)** Xylem cell area. **(F)** Number of phloem cells. **(G)** Phloem cell area. ** indicates extremely significant difference at P<0 .01.

The *35S*::*SlBL4*-RNAi silencing in transgenic tomato was achieved following the methodology detailed in Yan et al. (2020). The transgenic plants displayed up-curled leaves at the flowering bud stage, which contrasted with the normal phenotype observed during vegetative growth (**Figures 2A–D**). The curvature angle of the third to fifth fully expanded leaves at the middle stem position was approximately 10° in wild type (WT), compared to ~32° in the RNAi lines (**Figure 2E**). In the *SlBL4*-RNAi lines, the fruits exhibited a darker green color due to increased chlorophyll content (Yan et al., 2020). We also found that the leaves were darker green than those of the WT plants. Chlorophyll autofluorescence was stronger, and chlorophyll accumulation increased in the leaves of *SlBL4* RNAi lines compared to WT (**Figures 2E–J**). Further microscopic analysis of *SlBL4* RNAi leaves displayed fewer xylem and phloem tissues compared to WT plants (**Figures 3A–G**).

### Transcriptomic analysis of leaves between *SlBL4* RNAi lines and the wild type

**Figure 4:**
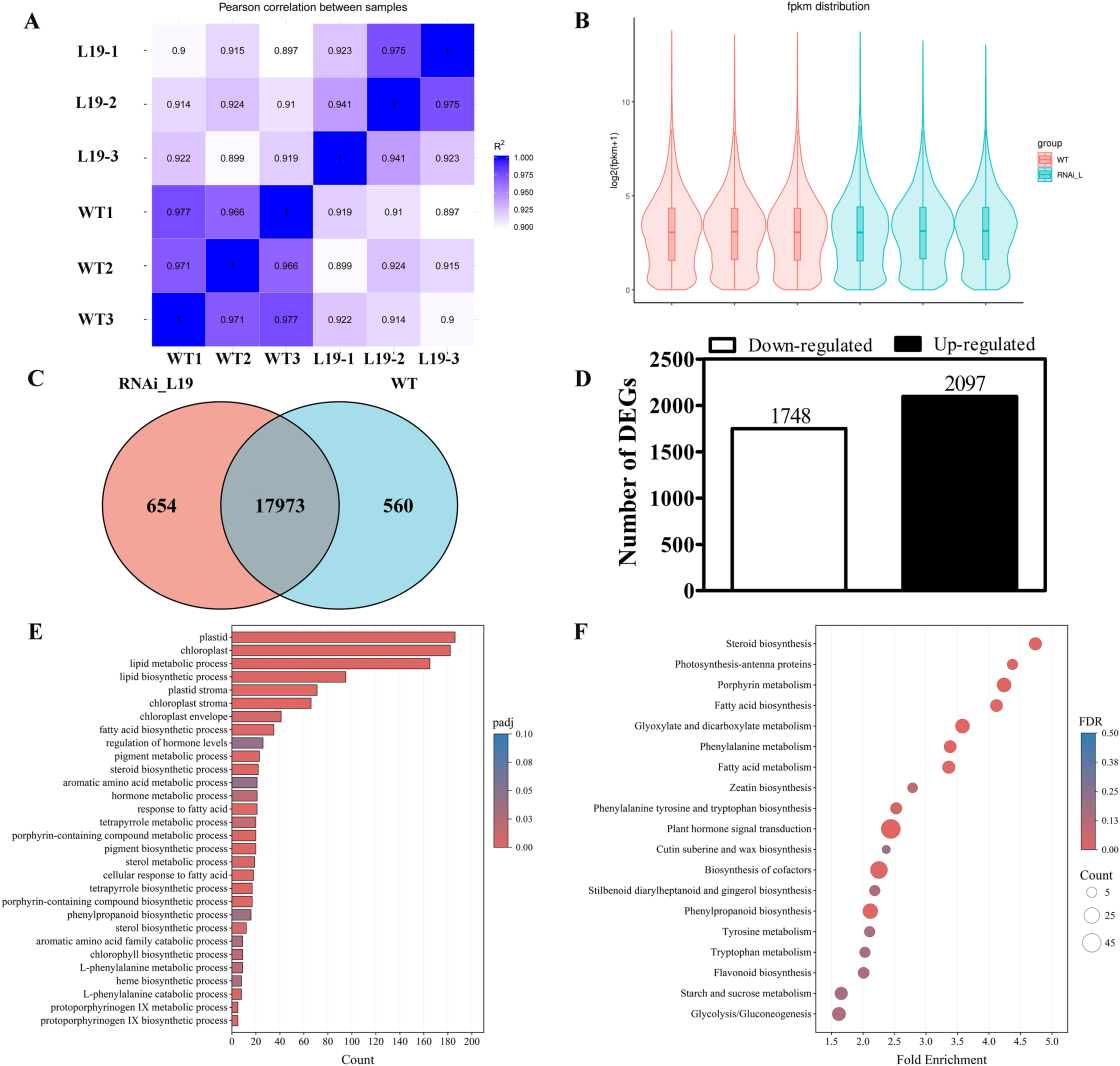
The DEGs of RNA-seq analysis of the tomato wild type and *SlBL4* RNAi L19. **(A)** Pearson correlation analysis of gene expression data from all RNA-seq libraries. **(B)** FPKM distribution of differentially expressed genes. **(C)** Venn diagram of the overlap and unique DEGs between WT and the RNAi L19. **(D)** Upregulated or downregulated DEGs at the 35-day developmental stages of the leaf. **(E)** GO function and pathway. **(F)** KEGG function and pathway.

To gain a transcriptomic understanding of the differences between *SIBL4* RNAi and WT plants, we conducted high-throughput RNA sequencing (RNA-seq) using Illumina technology on 35-day-old tomato leaves with three biological replicates. A Pearson correlation analysis and the distribution of FPKM values demonstrated that the RNA-seq data of RNAi-L19 and WT leaf samples were highly reproducible (**Figures 4A, B**). There were 17,973 overlapping genes and 1,214 DEGs between WT and RNAi L19 (**Figure 4C**). In the leaves of *SlBL4* RNAi plants, 2,097 DEGs were upregulated, and 1,748 were downregulated (**Figure 4C**, **Supplementary Excels S1**, **2**). Gene Ontology (GO) and Kyoto Encyclopedia of Genes and Genomes (KEGG) pathway analyses revealed that *SlBL4* knockdown affected multiple metabolic pathways, including hormone metabolism and signal transduction processes, the photosystem II oxygen-evolving complex, porphyrin and chlorophyll II metabolism, photosynthesis-antenna proteins, cell wall and lipid metabolism, starch and sucrose metabolism, and phenylalanine, tyrosine, and tryptophan biosynthesis, among others (**Figures 4D–F****).**

### Silencing of *SlBL4* affected the expression patterns of genes related to leaf polarity, auxin-related, and chloroplast development

Silencing *SlBL4* resulted in up-curled leaves from the initiation of the floral meristem, which was a normal phenotype and was maintained during the vegetative growth stage (**Figure 2**). In total, 12 DEGs were associated with leaf polarity development, including axial regulators *YABBY 1*, *2*, *4*, and *5*; *ASYMMETRIC LEAVES 2*; *WUSCHEL-related homeobox 1* and *4*; *Topless-related 4*; and *LOB* domain-containing *4*, *6*, *37*, and *41* (**Table 1**). Among 33 auxin-related DEGs were IAA-amido synthetase *GH3.1* and *GH3.2*; *auxin efflux facilitator*; *auxin response factor 4*, *9*, and *11*; *auxin-induced* gene; and *auxin-binding ABP19a* (**Table 1**). *SlBL4* was reported to be involved in chlorophyll accumulation and chloroplast development in tomato fruit (Yan et al., 2020). Chlorophyll accumulation was increased in the leaves of *SlBL4* RNAi lines (**Figures 2E–I**). Transcriptomic analysis identified 28 DEGs related to chlorophyll accumulation and chloroplast development (**Table 1**). Additionally, 20 genes encoding TFs were upregulated or downregulated in the leaf of *SlBL4* RNAi lines compared to the WT plant, potentially influencing leaf polarity development. These genes belong to various families, including TCP, NAC, homeobox leucine zipper protein, bHLH, and the MYB family (**Table 1**).

**Table 1:** Differentially expressed genes in the leaf of wild-type and *SlBL4* RNAi plants.

Gene ID	Annotation	Fold change (log2)
Leaf polarity development		
Solyc03g096140	Yippee-like protein	10.4
Solyc03g096150	Yippee-like protein	5.90
Solyc03g096260	Yippee zinc-binding/DNA-binding/Mis18	1.84
Solyc04g078650	WUSCHEL-related homeobox 4	0.98
Solyc03g116750	WUS-interacting protein 2	0.59
Solyc03g117420	TOPLESS; WUS-interacting protein 1	0.43
Solyc01g091010	Axial regulator YABBY 1	−3.65
Solyc11g008830	ASYMMETRIC LEAVES 2	−2.35
Solyc03g063140	LOB domain-containing protein 6	−2.29
Solyc03g096160	Yippee-like protein	−2.17
Solyc11g045530	LOB protein 4	−1.40
Solyc03g118770	WUSCHEL-related homeobox 1	−1.06
Solyc12g009580	Axial regulator YABBY 5	−0.85
Solyc11g071810	Putative axial regulator YABBY 2	−0.84
Solyc03g119530	LOB domain-containing protein 41	−0.74
Solyc02g092550	LOB domain-containing protein 37	−0.71
Solyc08g079100	Protein YABBY 4	−0.68
Solyc01g107190	AS2-like protein 39	−0.67
Solyc11g070140	Cell division control protein 2 homolog	−0.65
Solyc02g083540	Cell number regulator 13	−0.54
Auxin-related		
Solyc01g107390	Indole-3-acetic acid-amido synthetase GH3	3.13
Solyc10g079640	IAA-amino acid hydrolase ILR1-like 6	2.03
Solyc06g073050	IAA-amino acid hydrolase ILR1-like 5	1.13
Solyc05g006220	IAA-amino acid hydrolase ILR1-like 2	1.90
Solyc03g121270	IAA-amino acid hydrolase ILR1-like 1	0.95
Solyc02g062230	Auxin-responsive protein SAUR50	1.35
Solyc03g082530	Auxin-responsive protein SAUR32	1.81
Solyc03g082520	Auxin-responsive protein SAUR32	1.39
Solyc10g074790	Protein PIN-likes 6	0.71
Solyc02g037550	Protein PIN-likes 3	1.92
Solyc04g082830	Protein PIN-likes 1	1.66
Solyc05g056040	Auxin response factor 11	0.60
Solyc02g092820	GH3.1	−2.80
Solyc01g098550	Tryptophan synthase alpha chain	−0.39
Solyc03g112460	Tryptophan aminotransferase-related protein 2	−0.70
Solyc12g008690	IAA-amino acid hydrolase ILR1-like 1	−0.43
Solyc11g013310	Auxin transporter-like protein 3	−0.91
Solyc10g076790	Auxin transporter-like protein 2	−0.98
Solyc07g054580	GH3 auxin-responsive	−1.05
Solyc09g014380	Auxin transporter-like protein 2	−0.52
Solyc01g096070	Auxin response factor 9	−0.64
Solyc11g069190	Auxin response factor 4	−0.55
Solyc01g110605	Auxin-responsive protein SAUR50	−2.54
Solyc01g110590	Auxin-responsive protein SAUR50	−2.07
Solyc01g110600	Auxin-responsive protein SAUR50	−1.98
Solyc10g083320	Auxin-responsive protein SAUR50	−1.84
Solyc06g053260	Auxin-responsive protein SAUR32	−0.59
Solyc01g110730	Auxin-responsive protein SAUR24	−0.98
Solyc01g110660	Auxin-responsive protein SAUR21	−0.86
Solyc07g008020	Auxin-responsive protein IAA29	−0.91
Solyc12g007230	Auxin-responsive protein IAA27	−0.51
Solyc01g110940	Auxin-induced protein 15A	−1.71
Solyc07g041720	Auxin-binding protein ABP19a	−1.90
Solyc03g123410	Auxin-binding protein ABP19a	−1.88
Chlorophyll accumulation and chloroplast development		
Solyc12g005300	Chlorophyllase-2, chloroplastic	0.49
Solyc10g085040	Heme-binding-like protein	2.73
Solyc10g085030	Heme-binding protein	2.59
Solyc07g061800	Heme-binding protein	1.29
Solyc04g079730	Allene oxide synthase 1, chloroplastic	4.36
Solyc12g010020	Leucine aminopeptidase 1, chloroplastic	3.62
Solyc12g010030	Leucine aminopeptidase 2, chloroplastic	4.12
Solyc09g011870	Arogenate dehydrogenase 2, chloroplastic	2.47
Solyc06g050630	Arogenate dehydrogenase 1, chloroplastic	1.59
Solyc08g074620	Polyphenol oxidase E, chloroplastic; Short	2.01
Solyc04g079730	Allene oxide synthase 1, chloroplastic	4.36
Solyc07g039260	Granule-bound starch synthase 2	4.74
Solyc03g019660	Thylakoid lumenal, chloroplastic	−2.14
Solyc10g077040	Magnesium-protoporphyrin IX monomethyl ester cyclase	−0.50
Solyc10g008740	Magnesium-chelatase subunit ChlI	−0.45
Solyc03g118240	Magnesium protoporphyrin IX methyltransferase, chloroplastic	−0.45
Solyc10g007320	Uroporphyrinogen decarboxylase 1, chloroplastic	−0.79
Solyc09g092110	Light-regulated protein	0.61
Solyc12g009200	Photosystem I chlorophyll a/b-binding protein 6	−1.05
Solyc02g014150	Photosystem II stability/assembly factor HCF136	−0.38
Solyc09g064500	Photosystem II reaction center PSB28 protein	−0.68
Solyc06g060340	Photosystem II 22 kDa protein	−0.64
Solyc02g069455	Photosystem I reaction center subunit III	−0.92
Solyc12g009200	Photosystem I chlorophyll a/b-binding protein 6	−1.05
Solyc07g022900	Photosystem I chlorophyll a/b-binding protein 5	−0.56
Solyc03g062720	Photosynthetic NDH subunit of subcomplex B 2	−0.73
Solyc01g009990	Photosynthetic NDH subunit of lumenal location 5	−0.74
Solyc05g007780	Photosynthetic NDH subunit of lumenal location 2	−0.46
Transcription factor		
Solyc10g055760	NAC6	1.51
Solyc11g068620	NAC90	1.63
Solyc04g078670	NAC 19-like	0.89
Solyc02g081270	NAC71	−1.12
Solyc07g066330	NAC 21/22	−0.87
Solyc11g020670	TCP12	0.81
Solyc09g008030	TCP19	−1.08
Solyc12g014140	TCP3	−0.82
Solyc06g070900	TCP17	−0.78
Solyc07g062680	TCP2	−0.60
Solyc04g009180	TCP14	−0.50
Solyc01g096320	ATHB-12	2.96
Solyc03g082550	ATHB-7	1.44
Solyc05g051460	ATHB-6	1.07
Solyc11g010270	ATHB-5	−0.46
Solyc10g078670	bZIP-1	5.31
Solyc03g006910	bHLH 19	5.16
Solyc03g118310	bHLH 83	−2.50
Solyc04g079360	MYB44-like	3.22
Solyc07g052300	MYB101	−5.30

SlBL4 targets the promoters of *GH3.2*, *GH3.1*, and *LOB4* to regulate leaf polarity.

**Figure 5:**
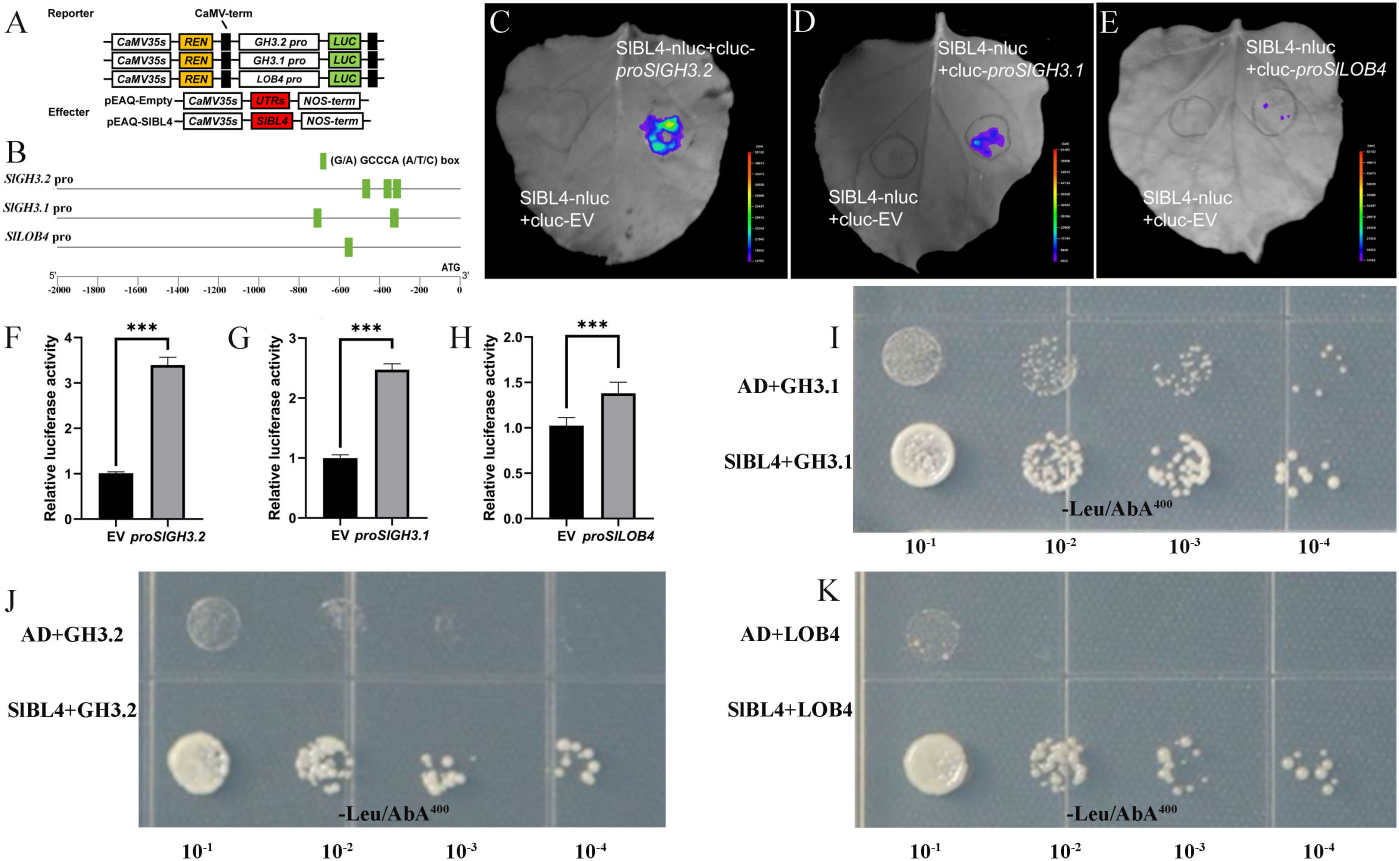
SlBL4 directly activates the expression of genes related to leaf polarity development. **(A)** Reporter and effector constructs used in the transient dual-luciferase experiments in leaves of tobacco seedlings. LUC, firefly luciferase; REN, Renilla luciferase. **(B)** The physical map of cis-elements in the promoter region of *GH3.*2, *GH3.*1, and *LOB4.* **(C–K)** SlBL4 biological effects on *GH3.*2, *GH3.1*, and *LOB4* transient assay promoters of tobacco leaf. In this format, the data are presented in the means (±SE), *n* = 6. Significant differences were determined with WT: Student’s *t* test, *p* < 0.001. **(H–J)** Yeast one-hybrid assay showing the binding of SlBL4 to *GH3.*2, *GH3.*1, and *LOB4* promoter. Transformed yeast cells were spread on -/Leu or -Leu/AbA media to screen positive transformants.

*GH3.2* (*Gretchen Hagen 3.2*) (Solyc07g054580), *GH3.1* (Solyc02g092820), and *LOB4* (*lateral organ boundaries 4*) (Solyc11g045530) promoter sequences were analyzed to identify the promoter binding sites of (G/A) GCCCA (A/T/C) boxes (Yan et al., 2020). The copy numbers of the SlBL4 binding motif in *GH3.2*, *GH3.1*, and *LOB4* promoters (2,000 bp upstream of ATG) are 2, 3, and 1, respectively (**Supplementary Table 1**, **Figure 5B**). Transient dual-luciferase assays were conducted to establish whether SlBL4 directly affects the expression of the *GH3.2*, *GH3.1*, and *LOB4* genes. Tobacco leaves were co-transformed with LUC reporter vectors containing the promoters of the *GH3.2*, *GH3.1*, and *LOB4* genes, and the effector vectors based on the promoters of the *SlBL4* gene (*CaMV35S*). The assays demonstrated that SlBL4 expression significantly increased luminescence readings with the *GH3.2*, *GH3.1*, and *LOB4* promoters compared to the control (**Figures 5A–G**). Yeast one-hybrid assays also confirmed that SlBL4 binds to the promoters of *GH3.2*, *GH3.1*, and *LOB4*, respectively (**Figures 5H–K**), indicating that SlBL4 activates the transcription of these genes.

**Figure 6:**
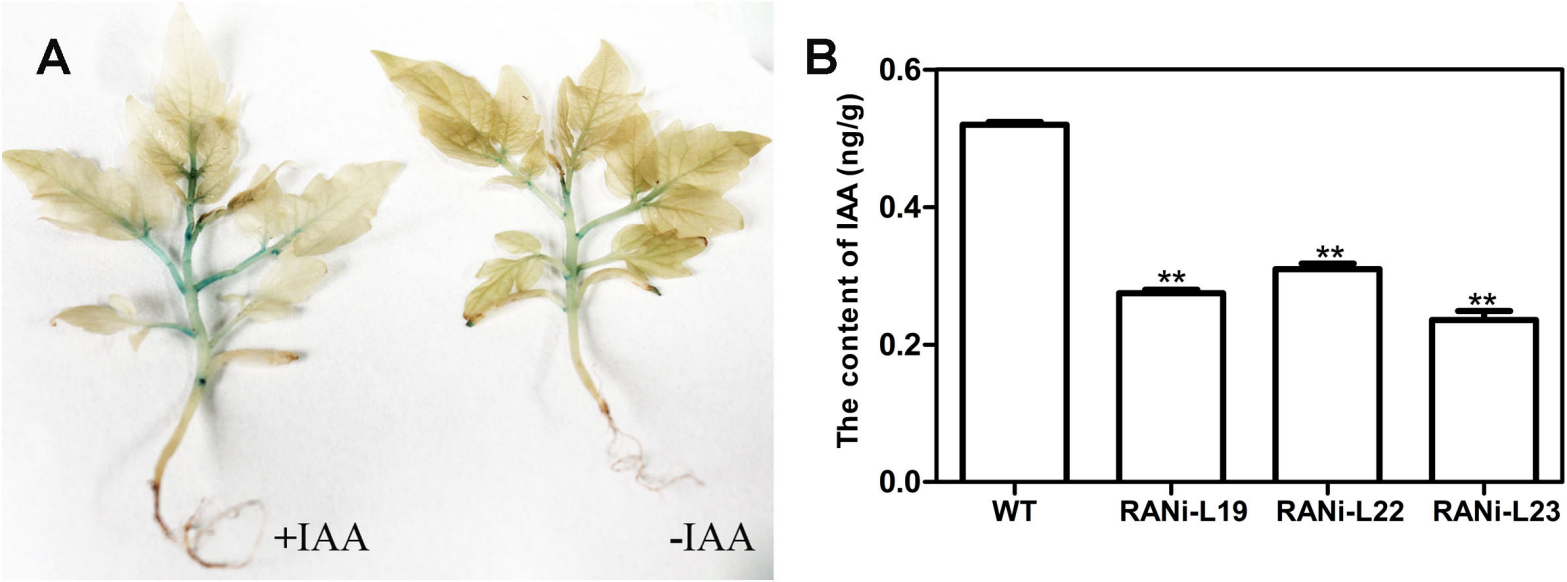
Effects of IAA treatment on the seedlings of *SlBL4*pro::*GUS* plants and auxin concentrations in the leaves. **(A)** Exogenous effects of auxin +IAA and control −IAA effects on 2-week seedlings of *SlBL4*pro::*GUS* plants. **(B)** IAA contents; the standard errors are indicated by vertical bars. The asterisks denote the vast variations at *p* < 0.01 obtained through the *t* test assistance of *SlBL4* RNAi plants among three lines; WT, wild type; RNAi L19, RNAi L22, and RNAi L23. ** indicates extremely significant difference at P<0 .01.

To investigate whether *SlBL4* downregulation modulates auxin sensitivity, we analyzed the root phenotypic responses of the transgenic lines to exogenous IAA treatment. Compared to WT seedlings, the *SlBL4* RNAi lines exhibited significantly shorter primary roots following IAA exposure, and a marked reduction in lateral root numbers was observed in these RNAi lines upon treatment with 0.5 and 1.0 μM IAA (**Supplementary Figure 2**). Additionally, ultra-performance liquid chromatography (UPLC) quantified endogenous IAA levels in tomato leaves, showing significantly lower IAA concentrations in *SlBL4* RNAi lines compared to WT plants (**Figures 6A, B**). Overall, these results indicate that silencing *SlBL4* affects auxin signaling, polar transport, and endogenous content in tomatoes.

## Discussion

The *BELL* TFs have been reported to play diverse roles in plant morphology, fruit development, and fruit pedicel abscission (Hay and Tsiantis, 2010; Yan et al., 2020; Yan et al., 2021). However, their involvement in establishing leaf polarity remains largely unclear, especially in economically significant crops such as tomato. In this study, we provide molecular and phenotypic evidence demonstrating that *SlBL4*, a tomato BELL-type homeodomain TF, is a critical regulator of leaf polarity patterning.

The qRT-PCR results revealed that *SlBL4* transcripts were significantly enriched in leaf tissues, implying a tissue-specific function during leaf development. Repression of *SlBL4* by RNA interference resulted in pronounced upward leaf curling, altered epidermal cell differentiation, and rearranged vascular patterning (**Figures 2**, **3**), which are typical indicators of altered leaf polarity. These morphological defects suggest that SlBL4 is essential for maintaining proper leaf symmetry and structural integrity in tomato. Collectively, these observations support the notion that BELL homologs across angiosperms may share a conserved function evolutionarily in regulating leaf development and polarity establishment, extending the functional scope of BELL TFs’ previously reported roles in reproduction and organ maturation. To further dissect the underlying molecular network, we performed transcriptome sequencing to identify genome-wide gene expression changes triggered by *SlBL4* downregulation (**Table 1**). The results revealed that downregulation of *SlBL4* significantly altered the transcript abundance of numerous genes implicated in transcriptional regulation, hormone signaling, cell differentiation, chlorophyll accumulation, chloroplast development, and leaf patterning. A total of 65 genes exhibited significantly differential expression in transgenic plants and may be related to the development of leaf polarity, including genes from the *Yippee-like* family, *WUSCHEL*-related, axial regulator *YABBY*, *ASYMMETRIC LEAVES 2*, *LOB*, *hormone regulator* (auxin synthesis and transport), and TFs (*TCP*, *NAC*, *bHLH*, and *homeobox leucine zipper*) (Siegfried et al., 1999; Guo and Gan, 2005; Tanaka et al., 2012). Notably, key regulatory genes that define adaxial cell identity, particularly those within the AS/LOB family, exhibited significantly altered expression in *SlBL4* RNAi lines. Meanwhile, *YABBY* family genes, which specify abaxial cell fate, were markedly upregulated in transgenic leaves compared to WT plants. These expression changes align with the abaxialized or adaxial–abaxial imbalanced phenotypes observed in *SlBL4*-repressed plants, indicating that SlBL4 functions upstream of the core genetic hierarchy governing leaf polarity. In addition to transcriptional regulators, auxin metabolism, transport, and signaling constitute a fundamental module that reinforces robust leaf polarity and demarcates the boundary between adaxial and abaxial domains (Xu et al., 2007; Tameshige et al., 2013; Wang et al., 2022). In this study, multiple auxin-related genes, including those encoding auxin biosynthetic enzymes, transporters, and auxin-responsive proteins, showed substantial differences in expression in RNAi plants (**Table 1**). These results suggest that SlBL4 modulates leaf polarity partly by shaping local auxin homeostasis and distribution. Future investigations may focus on the dynamic distribution of auxin in leaf primordia and the precise contribution of SlBL4-dependent auxin gradients to polarity establishment. Furthermore, downstream gene mutants were created using the CRISPR/Cas9 gene-editing system for functional characterization (Hu et al., 2019; Hu et al., 2025). However, further experiments beyond the scope of this study are needed to determine the relative significance of *SlBL4* and these genes in establishing leaf polarity.

Proper auxin distribution and homeostasis are crucial for maintaining adaxial identity. In symmetrical auxin distribution, localization may occur on either side, implying that a lower concentration of auxins on the adaxial surface is necessary to maintain, or at least not inhibit adaxial identity. The regulation of leaf polarity gene transcription by *SlBL4* is not yet fully understood. It is hypothesized that *SlBL4* performs its developmental functions through direct transcriptional control of downstream targets, which in turn modulate auxin signaling and polarity-related gene networks. Several transcriptional networks are involved in the process of communication of the auxin signal in relation to the leaf polarity during development (Xu et al., 2007; Bowman and Floyd, 2008; Husbands et al., 2009; Braybrook and Kuhlemeier, 2010). The IAA-amido synthetases (GH3) contribute to converting the IAA to IAA–amino acid conjugates, and they are part of the intracellular auxin homeostasis (Böttcher et al., 2010, Böttcher et al., 2011; Kumar et al., 2012a). Using yeast one-hybrid and dual-luciferase reporter assays, we demonstrated that SlBL4 directly binds to the promoters of *GH3.2* and *GH3.1*, and activates their transcription (**Figure 5**). We therefore propose that dysregulation of auxin homeostasis caused by reduced *GH3.2* and *GH3.1* expression partially contributes to the upward-curling leaf phenotype in *SlBL4* RNAi plants. Members of the LOB (LBD) domain protein family also play roles in various aspects of plant organ boundary formation (Rong et al., 2024). For instance, AS2 (LBD6) regulates leaf proximal–distal axis patterning by repressing *KNOX* genes and restricting cell proliferation in the axial region (Iwakawa et al., 2007). In this study, SlBL4 was found to directly induce the expression of *LOB4* (**Figure 6**), further supporting its role in fine-tuning the expression of genes essential for leaf polarity. Taken together, these results suggest that *GH3.2*, *GH3.1*, and *LOB4* function in an auxin-related pathway dependent on SlBL4 to govern leaf polarity. Our findings reveal a direct regulatory module in which SlBL4 physically targets and modulates the expression of *GH3.2*, *GH3.1*, and *LOB4*, thereby coordinating auxin homeostasis and transcriptional programs for proper leaf patterning. A key direction for future research will be to elucidate the initial establishment of adaxial–abaxial gene expression patterns in very young leaf primordia and to identify additional SlBL4-interacting proteins that may modulate its transcriptional activity. Further studies will also help clarify the functional divergence and specialization of this unusual BELL protein in tomato development.

**Figure 7:**
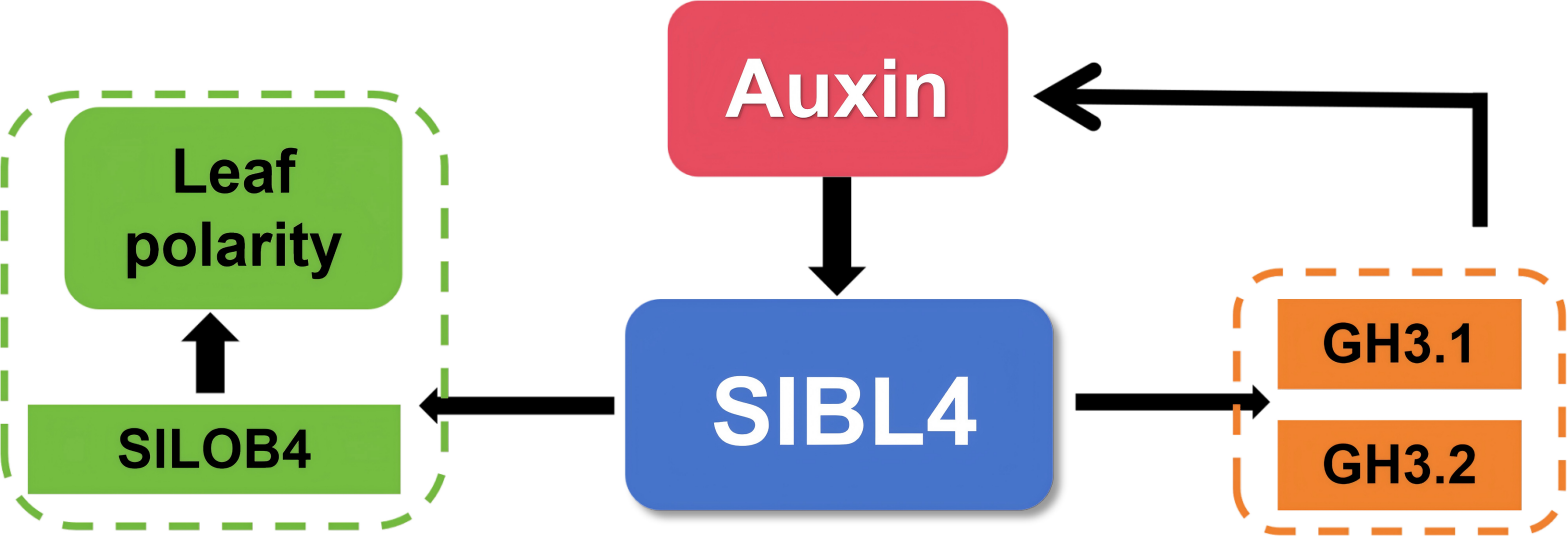
The role of SlBL4 in tomato development.

Previous studies demonstrated that SlBL4 regulates tomato fruit development, including chlorophyll accumulation, chloroplast development, and cell wall metabolism, by directly binding to the promoters of *SlPPO*, *SlCHLD*, *SlPOR*, *CABs*, *TKN2*, and *SlPE*, thereby repressing their expression (Yan et al., 2020). Additionally, SlBL4 directly interacts with the promoter of *LeSPL*-*CNR*, *JOINTLESS*, *OVATE*, *LAX3*, and *PIN1* (Yan et al., 2020; Yan et al., 2021). In this study, our results reveal that SlBL4 binds directly to the promoters of *GH3.2*, *GH3.1*, and *LOB4* and positively regulates their transcription. This interaction modulates the expression of these core regulators to control leaf morphology and adaxial–abaxial polarity (**Figure 7**). Collectively, these findings establish SlBL4 as a multifunctional master regulator that orchestrates diverse biological processes throughout tomato growth and development, including chloroplast biogenesis, fruit ripening, pedicel development, and leaf polarity patterning. The identification of the SlBL4-*GH3.2*/*GH3.1*/*LOB4* module not only deepens our mechanistic understanding of leaf polarity control but also offers a valuable target for the genetic improvement of plant architecture, stress resistance, and crop productivity. This study expands the functional landscape of BELL homeodomain TFs and provides novel insights into the transcriptional and hormonal integration that governs organ polarity in *Solanaceae* crops.

### Plant material, growth conditions

Micro-Tom (Tomato) was grown under greenhouse conditions: 16/8 h of light/dark (25 ± 2)/(18 ± 2), 70%–80% humidity, and intense light of 250 µmol m^−2^ s^−1^. Roots, stems, and leaves were collected from 2-month-old plants; the flowers (at bud and anthesis stages) and fruit (at immature, breaker, breaker+2, and red fruit stages) were harvested at their respective developmental times. In the case of plant hormone treatments, 5-week-old seedlings were treated by means of 0.1 mM solutions of KT, NAA, ABA, GA_3_, ethylene, D-Mannitol, NaCl, SA, and meJA individually after every 2 days over a period of three treatments. Untreated leaves were used as the control since the leaves were harvested after treatment. The leaf divergence angle was measured by image analysis. The vertical plumb line of the main stem was set as the 0° reference. Each leaf was bisected along the midrib into adaxial and abaxial sides, and the divergence angle was defined as the angle between the midpoint of the abaxial leaf margin and this vertical reference. For each group, at least six plants were randomly selected. The third to fifth fully expanded leaves at the middle stem position of each plant were measured. All angle data were acquired using ImageJ software and are expressed as mean ± standard deviation (SD).

### Expression analyses by qRT-PCR

For the expression analysis of *SlBL4* in tomato, roots, stems, leaves, flowers, and fruits at various stages of maturation and ripening were collected, immediately frozen in liquid nitrogen, and stored at −80 °C for RNA extraction. Total RNA was extracted using the TRIZOL method. First-strand cDNA synthesis was conducted with the Fermentas reverse transcription kit (Fermentas, UK). qPCR was performed according to the manufacturer’s instructions (SoFast™ Supermix, Bio-Rad). The sequences of the primers are provided in **Supplementary Table 1**.

### Generation of transgenic tomato plants

The RNAi fragment was cloned from the cDNA of tomato (*Solanum lycopersicum*) cv. Micro-Tom using the forward primer 5′-ATGGGAGAACATTTCAACAA-3′ and reverse primer 5′-GACTCCTTGTCAATCCAC-3′. For RNAi vector construction, the 253-bp sense and antisense fragments of *SlBL4* were amplified separately. The sense fragment was digested with restriction enzymes *Eco*R I and *Kpn* I, while the antisense fragment was digested with *Hind* III and *Bam*H I (New England BioLabs, Ipswich, MA, USA). The digested fragments were cloned into the pHANNIBAL vector sequentially. The resulting *SlBL4*-RNAi expression cassette was then inserted into the plant binary vector pCAMBIA2301, which had been pre-digested with *Sac* I and *Spe* I (New England BioLabs). Tomato transformation was performed using *Agrobacterium tumefaciens* strain GV3101. Transgenic RNAi lines were initially selected on medium containing 100 mg L^−1^ kanamycin. *SlBL4*pro-*GUS* plants were acquired based on their approach in a prior research (Yan et al., 2020; Yan et al., 2021). This study has chosen transgenic lines 19, 22, and 23 (T_3_ generation) to be further analyzed and WT Micro-Tom tomato as a mock.

### Chlorophyll content and chlorophyll fluorescence analysis

To measure the content of chlorophyll, the leaves were taken at 35 days and analyzed as per Powell et al. (2012). Observations of chlorophyll autofluorescence were done using Z-series confocal laser-scanning microscopy (Leica, Germany).

### Anatomic characterization, microscopy, and GUS staining analysis

To study the histological evidence, the leaves and the young shoots of the WT and RNAi plants were collected at 35 days old, placed in the FAA solution at 25°C and stored for 72 h, and subsequently dehydrated in ethanol series and embedded in paraffin. Images were acquired using a Nikon inverted fluorescence microscope equipped with a 10× objective, and a scale bar of 50 μm was applied. Subsequent quantification of the number and area of xylem and phloem cells was performed with ImageJ software. Cell counts and area measurements were repeated three times per field of view to obtain a consistent average for each sample, and the data are presented as the mean ± SD. Each line was analyzed using six biological replicates. In their article, Yuan and others mentioned the GUS staining technique (2018) (Yuan et al., 2018). Leaf, stem, root, flower and fruit tissues of transgenic plants were sampled at 45 days after anthesis, respectively.

### Auxin treatment and auxin content measurement

To treat the seedlings with IAA, the seedlings were placed in a 1/2 MS layer of 50 μg/g IAA for 24 h, and subsequently, the seedling explants were stained using GUS following the procedure by Yan et al. (2021). The same was repeated, and 10 leaves were taken at the exact location to quantify IAA as per the literature with minor amendments (during sample extraction, centrifugation was performed at 13,000*g* for 15 min) (Ma et al., 2015).

### RNA-seq analysis

RNA-seq analysis involved the collection of the leaves (35 days) of WT and *SlBL4* RNAi (line 19) plants. The total RNA was extracted with the Trizol agent (Invitrogen, USA), and libraries of the RNA-seq were prepared and sequenced on an Illumina HiSeq 2000 at the Novogene Technology Co., Ltd (Beijing, China). After quality filtering, clean tags were obtained and mapped to the annotated genome sequence in the Tomato Sol Genomics Network database (http://solgenomics.net/). The number of tags for each gene was normalized to tags per million (TPM) to generate the gene expression matrix. Differentially expressed genes (DEGs) between the RNAi line 19 and WT samples were analyzed following the method described previously (Audic and Claverie, 1997). Genes with a false discovery rate (FDR)≤0.001 and an absolute log2 ratio≥1 were used as thresholds to determine the significance of differences in gene expression.

### Dual-luciferase transient expression assay

The entire coding frame of *SlBL4* was amplified and later inserted as an effector vector into the pEAQ-Empty. The reporter vector was made by cloning the promoters of the *GH3.2*, *GH3.1*, and *LOB4* genes into the pGreenII 0800-LUC vector. *SlBL4* transient expression was done in tobacco leaves (*Nicotiana benthamiana*). Yan’s method used a dual-luciferase assay kit to measure the LUC and REN luciferase activities (Promega, USA) (Yan et al., 2020). A total of six biological repeats were done, and primer sequences are provided in **Supplementary Table 1**.

### Yeast one-hybrid assays

The full-length coding sequence of *SlBL4* was amplified and cloned into the pGADT7 vector. Promoter fragments of *GH3.2*, *GH3.1*, and *LOB4* were inserted into the pAbAi vector, and the resulting recombinant plasmids were transformed into the Y1HGold yeast strain in accordance with the manufacturer’s protocol for the Yeastmaker™ Yeast Transformation System 2 (TaKaRa). The pGADT7-*SlBL4* plasmid or empty pGADT7 vector (as a negative control) was individually transformed into the aforementioned recombinant yeast strain, and the transformed yeast cells were plated on SD/-Leu or SD/-Leu/AbA (400 ng/mL) selective media (Clontech, San Francisco, CA, USA).

## Data Availability

The data used to support the findings of this study are available from the **Supplementary Material**, and raw sequencing datasets of RNA-seq are available in the National Center of Biotechnology Information (NCBI GEO: PRJNA1428830).

## Supplementary material

Supplementary Figure 1 The expression of quantitative PCR analysis of the Solyc01g107390, Solyc03g006910, Solyc07g054580, Solyc02g092820, Solyc11g045530, and Solyc07g039260 in tomato leaf in RNAi plants (L19, L22, and L23) compared with the WT plant; The quantitative PCR data represent mean values for three independent biological replicates. Asterisks indicate statistical difference of the value at the P < 0.05 (*) levels as determined by t-test; Standard errors are indicated by vertical bars.

Supplementary Figure 2 Root development was assessed in two-week-old wild-type (WT) seedlings and three independent *SlBL4* RNAi lines (L19, L22, L23) grown on half-strength MS medium supplemented with different concentrations of indole-3-acetic acid (IAA; 0, 0.25, 0.5, and 1.0 μM). Scale bars: 1cm (Yan et al., 2021).

Supplementary Table 1 The primers used in this study.

Supplementary Excel S1 Up-regulated differentially expressed genes in wild type and *SlBL4* RNAi plants of leaf development.

Supplementary Excel S2 Down-regulated differentially expressed genes in wild-type and *SlBL4* RNAi plants of leaf development.
