# Supplementary figures and images for: SlBL4 is involved in leaf polarity development in tomato
Source: Front Plant Sci. 2026 Apr 28;17:1765515. doi: 10.3389/fpls.2026.1765515 (PMC13161086; doi:10.3389/fpls.2026.1765515)

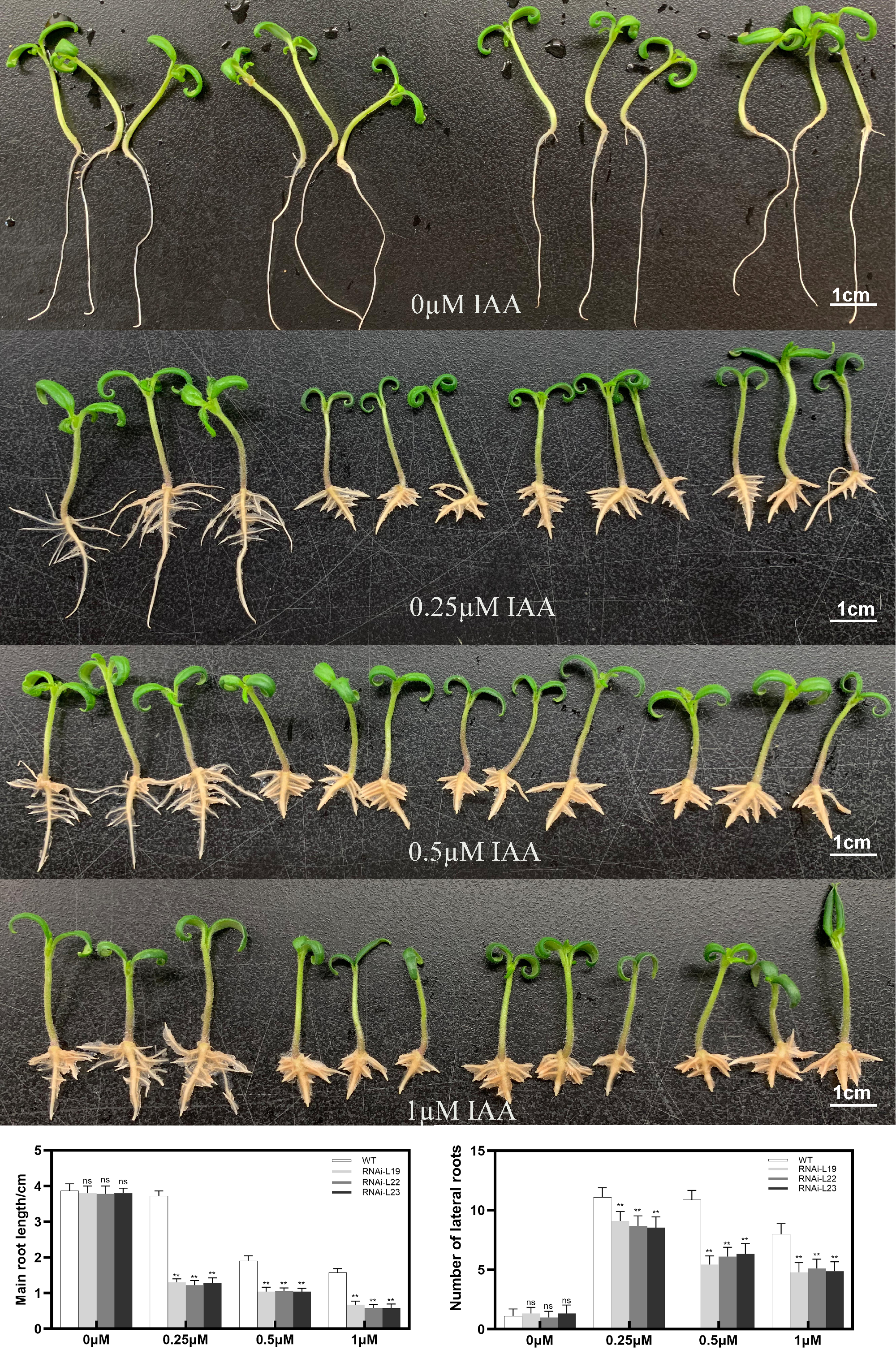

Supplement: Supplementary Figure 2 — Root development was assessed in two-week-old wild-type (WT) seedlings and three independent SlBL4 RNAi lines (L19, L22, L23) grown on half-strength MS medium supplemented with different concentrations of indole-3-acetic acid (IAA; 0, 0.25, 0.5, and 1.0 μM). Scale bars: 1cm (Yan et al., 2021). [file Image2.tiff]
