# Supplementary material for: SlBL4 is involved in leaf polarity development in tomato
Source: Front Plant Sci. 2026 Apr 28;17:1765515. doi: 10.3389/fpls.2026.1765515 (PMC13161086; doi:10.3389/fpls.2026.1765515)
Supplement: Supplementary Excel S2 — Down-regulated differentially expressed genes in wild-type and SlBL4 RNAi plants of leaf development. [file Table3.docx]

Table S1 Primer sequence associated with this article experiments.

| Primer | Primer sequence (5’ to 3’) | Orientation |
| --- | --- | --- |
| *qSlBL4-F* | AGCAGTGGGCACTTTCAGCAGC | Sense |
| *qSlBL4-R* | CTGGCAAGCCTCTTTGTGGCCT | Antisense |
| *SlUbi-F* | GCCGACTACAACATCCAGAAGG | Sense |
| *SlUbi-R* | TGCAACACAGCGAGCTTAACC | Antisense |
| *qSolyc01g107390-F* | ACCGGAGATTCAACGGATCG | Sense |
| *qSolyc01g107390-R* | TTTCACCAGCTGACGTTCCA | Antisense |
| *qSolyc07g054580-F* | TGCCCTTTACCCACCATAGC | Sense |
| *qSolyc07g054580-R* | AGTGTGTTGTGGCTGTTCCT | Antisense |
| *qSolyc02g092820-F* | ACCGCGATTGAAAATGCGAC | Sense |
| *qSolyc02g092820-R* | GGGTTAGCCGGGTCCTTAAC | Antisense |
| *qSolyc11g045530-F* | TGGCAATTGCACAAGCTGAA | Sense |
| *qSolyc11g045530-R* | AAGGGACATGGTTGGCTGAG | Antisense |
| *qSolyc07g039260-F* | TTCAAGCTGTGTGAGCCAGT | Sense |
| *qSolyc07g039260-R* | TGATGCAATCTCCAACGACCA | Antisense |
| *qSolyc03g006910-F* | CGCGAACTCATATTCCTCCGA | Sense |
| *qSolyc03g006910-R* | GAGCGCAGCCATTTCTTGTC | Antisense |
| *ProSlGH3.2-F* | ATGAGCGAGTACATTAAATT | Sense |
| *ProSlGH3.2-R* | ATTTTGCTAGGCATGCAGCC | Antisense |
| *ProSlGH3.1-F* | TATCTTTTCATTCGACATGAAGC | Sense |
| *ProSlGH3.1-R* | TATTTAAAAAAAGTGGAAGTGAG | Antisense |
| *ProSlLOB-F* | AGTTTGCTCTCCAGATCCATG | Sense |
| *ProSlLOB-R* | TTTACCTATTTACACACAACTC | Antisense |

**GH3.2 promoter Solyc07g054580**

ATGAGCGAGTACATTAAATTTGTGACCTCAAAGTCTTTGGTAGTACACAATTATACACAATCAAGATGATTATGCAACTTCAATTTCTTTGAAGAATGAAATGAAACTTTTCGATTTTTTCTTTAGTGTTTGATATTTGTGTTAGAATTTGATTACTTCTGAATATACACTTAAAAGTCTCATATTAGGAATAAAGTGCTCCCTAACAAAGCAAATTTCGTATTCAAAAAGACTCAAATCTAAAATATCAAATTACAAATAAAAGAATACTCACCAACCATTCCATCACAATATGCGTCCATCACTACTTTGAAAAATATCCATTTTACTTATTAGACGTAAAATAATAAATAATAATTGGTCCCCTCTATGGACAACCACTTTTTAGTCTTTAACCTTAAACGTTATCTACTGTGCTAAATCACGAGCCTACTCAATACAAAGATAATATGCATGTGTACCAGGTTCATTTGATTTTTTACTTTTATTTTTTGTACAAGAAAAATAAATTTATGTGTAAAAATCTATTCATTTAAAAAATACAATTACAAACTTCCTATTTTTATTTACTTTGGGCTTAGGCCTACATAGATTTGATTTTTGGATTTTTCCCATTTCAATATGTGATTTCGCCTAAGGTGTAATGTGTATCCTTTTTTCAAAGGCTTGTCCTCCGTCAATTACATGGTCGTCACAAATTCATAAAATATTTGAGCTTATAAAATTTAAATCATGGATTCGATCGCCTCCATGGACACTTCTTTAATTTTCTTTTAAGTTCACCATTTAAGATTTATTGGTAAATAAAAATAGGAAGTTTTTGATATATATTTATATAGATAAACATCCTATTTTGACTTTCATTGATACGAATTATAATAAAAAATAAAAAAGACTTTTGAATCTTGTAATCTTTTTATTAAACATGTCGTTTACGTGTAAAGTTGAAATGAAAGAGTTTGAAAAGGGATTTTTTTAAACAGAAAATAGGATAAACAAATTGAAACGAAGAAAATATATAACATATATAGCTGTTAAATTTATGATTCTTGGTCCCTACATCATATCTACTATAGATACTCTTATCTCCATCGAATTCCTAAAAGAAATGTGATGAAAGAAAAATTCTATGGTCCATAAAGAATTTGTACACATTTCTTTTAAAAGAGATACGGGGGTTGCTCTATTTTTGGATCATCAAATCACAATAATGCTTACAAAAATGATATTTCATAATGCATGTGGGCTGCATAATGCAGCAACTTGATACCCTATATATGTTTGTATTAATAATATAATTCTAGAAAAGGTAGATTATCGGAGTTTGAATAAATTATTTATTAATACATATATATAAATAAGATCAAAATCAAAATTATTGGATTCTGATAAACTCTGTAGGTAAAACTATAAGTTAATTTAATGTCCTTAACCGATGATGAAAACAGAATCAAGATTTCAACTAATAAGATGATTCAGATTATAAAAAAGTAAACATAGATTGAAGTAAAAAAAAGATTTAATATCAACTATATGTACGTTTTTCATTACCCCACGCACACCCTCATTTCGTTATTAGTTCATTTAGAAAAGAATAGCGTATTCTTATATTTTCATTGATAAGAAGTTTTTATATTTATAGTTATAGTAATATACAAATCTCCAACTCTTTATTATTGAATTTAGTGTCCAATCAAAACTATAATTTAAGTGAAACCTAAGTAATATCATCTTAATAGATGAAGTAAGTATAATTTTTCCAATATAAGTGAGACTTTAAAACTTTGATGTTGACAGCTAGCTTCTAAATAATAATTCTAATATAAACAGTTGTCGAGTCATGTGTAGTCAATTTTTAGAAAAGCCAAACAAATAATACAAATCATTTCCCAACAACAACAACATTATATTTATTATATAGTATATAGTTTTTTTTTTTTCTAACAACATTACATTACATATATACAATTTCTTCCTGCT GGCTGCATGCCTAGCAAAAT

**GH3.1promoter Solyc02g092820**

ACTTGAATTATTAATATCGTGAGTAGAAAGGATTCTACCAAAATCGTGTAACATAATAAACAATGAGAAACATGATATATTCGCACGCGCACACACACAATTAGAAAAAGATCAAAAAAAGGTTAACTGCCTGATTTGAAAAAATGTATGATCATCTCCTACCTGATTTGTTCTTATCTGAAAACAAACTGATTCTTTATATTGTTTTCTTTTACTAATATATTTATATGGGTCCTCTCTTCGTGTCAAGAATTTTAGTGTTATGGTCAAAACAATTTAGTTTCTAGAAATGAGAAAAAATAATTTTGGGTCTAACTCAATTAAAAAAGTTAGAAGAGAATTTCATAAGTCCGGCCATATAAGGAGACCGACCACTCATCATTTTTTTTTTATTCGAACACTTCAACACCCACCTCACATGTAAGACTAGATATCTAGTGTGTAGATCCAATTTAAAATGATTGTGTGATACCATATAAAGTGATAGACCTAAAAGCCTAACTTTAACGTTAAAAATTAACTTGTGATGAAAGAATGCCCAAATTCAAAAAGAAAATTACTCTATCTTTTCATTCGACATGAAGCTATTCAACATAGATTCAAGTTATTGATTATTTAAAAGAGTTACGATGCATACTGAAATTAGTGCTATACTATGGAAAAATCTTTTATCATTACTTTGGGAGTGGGAAAGAGAATATCATTGCTACATTACTAAACCTTTCACTTCAACTGTGGAACATATAAAACCCACCAAAATGAGTTAGAGGGTTAGGTATCAGAAATTATTTCATGAACCATGTTAAAATCGGCGCAAGTGATGGTAGGGAGTTGAAATATGAAACACAACCCACGTTAGAATATGAAAAAGTTCAACGAAACCCCTATGTAAAGCAAATCATATTGTAAAATTAATTAAACTTTAGTAATTCAATCAACAATATAACAAAAACAAATAAGGAAAACTATACTTATAGCCAAGAGAACCAAATGGGTTGGATCAAAATTCAGACGTGTCAAAATAAATTGAGTGAATAAACCATTCAAAAGTTATTTGCGGTAATTTAATCTTAATAAATTCATTTATAAAAATATCTAATAATCATTTTTCCTTTGTGATAACTATAAATAATTAATATATCAAATAAAAAATATCTTTCCAAAATATTATAGGAGATTGAATTACTCATTAATCAATCTCGCCTAAATATCAATTAAACTTTTACATAATTCAAAAATTAATTATTAAATAAGTAAATCATTAAAGAGTTAATGGATCATGATG**ATGGGCT**GATTTTGTCACTCTTAGCCGACAAGGAATTGACAAGGCGATGAAAACAATGGCAGGGGCATCCTGGTAGAAAGCCCGGGAGCTATACTACTAGACAATTAACTAAACAACAGTGATGAACCCGAGGATGCTCATGTGATTGCTGCAAAAATATATTCTTTAACTTTACATATTATATCAATTCTCCATAAAATTCTAACAATAAATAAACTATTTAACGGTTAATTAAGTTAATATTAGCTCCTAATAAATAGGCATTTTTTAATTTCAGTGAAATTTTGCCCCTTCGCCGGCAGCCTAATATTTGTCCGTGACCAACAAACTCACAGGAAAAGACAAAAAAACCCTAGCTGGATATTGCTGACCTGTCAAACAAAGTGGGCCCATTGGTTCTGAGTTGAGCAGACAAATGACGTTTTATGGGGTCCACATGCTCCCTCTTCAGTGGACCGTATACCCGACAAAAAATATTACAAAGTCGATGCACGGCAATGCTTCGTCCAAGTCCATGCCTAAAATTACCAAATATTTTTATTACTATGGTTTTCCACGTGTACATGCTTACGTGGAAAGTGACGTGGTAAACATGGAAGATTAAAACCTTCCTTTTACTATAAATAGGTTCCCTTCTTCAGCAACCAAACCACACAATTGAAATAAACAATAATCTTATTCTGCTTTAAATTCTCACTTCCACTTTTTTTAAATA

**LOB4promoter Solyc11g045530**

TGTGAATTGGTGAACTGAAAGGAGATTGGTTTTAATAGAAGGGGCGTGGGTAAGGTTACCTAGGTGAAAGATAGCGGTGGGGGTTTTAATGGTACCCGTGCCAGTGTGACAAATATTAAGGGACTCACCATTGCCAACAGTAATACATTGGAGCCATGATATGGATTTGGAGATTTAAGCTTGGATAGATCAGAAGTAACGTGCATGTTGGCCCCAGTATCCAACAACCATTCAGAGGAAGGGCCGGCTTGAGATGCATAATTGACACGGTTATCACTATTTCGGCTCTCCTCATACCGAAACCAGCAATGAACAGTGGTGTGTCCTATTTTCTGACAGATTTGACAAGTTGGACGATCCCGCTCGTAAGTGCCCTGCCCGCCGCTGGAAGATGACTGGCCGCCGCTGCCGAAGGAGTGCAGGCTGTTGTTGCTGCTGTGCTGCTGACCGTCGTATGGAGGACGACTGCCCTGCCGACCGCCGCGCCCGCGGCCTCCGCTGTTTTTGCCGTAGCCGCCGCGGCTCCCCTGCCGGCCGCCACCACGCCCCCCGCGATAGTTCTGGCTTGCGGTGAGGACGGTAGCCGGCTCCATAACACCGGCTTCTCAGAGAAGAAGTTTGCTCTCCAGATCCATGTTGATTTCTTCACTTTTTAGCCACGAGGAGAGAGTAGCCAGGTCAACGGGCGTTGGACTGATGCGAACCGCCTGTTTAATGGAGGAGTAAGCTGATGGGAGCCCCGGAACGACGCACATGACGAGATCTTTTTCGAGAATGATTTCGTTCACGGTGTCGAGGGCTGTGATGATGGTGGAGACCTCGTCTAAATACTCCGCCATAGTTTTCGTGCCTTTGGTAATTGTGTGGAGACGATCACGCAATTGAAAAATATGAGAGTGGGAAATTGATGCATAGCGTGTTGCGAGGGCCGTCCATAGGGCTGAAGCAGTTGTGTAGGATCGGACGTGTTTCTGAACCGTGGGAAAGATGACCGCAATCAAACATGATCGGACCTGGCCATCCACGGCTTTCCAGGCGGC**ATGGGCC**GGACTGTTTTTTTCTGATTTGTCCGTGGCGGTGATGACTGCCGGCGGCGGTTCTGTGCTTCCATCGACGTATTTGAGAAGGTAATTGGCCTCAAGGGCTGTAAGAACGGTGATCGGGAATCAGGGCATGAAGATGCCTGAGAAGGAGTTTAACGCCAGAAGGAAGTGAATCGAGAGGATCCACCTCGGTTGTCATGGCTGCCGCCGCCCAAGAGAAGAGAGGGAACGATCGGTGCCCTAAAGAGAAAAAAAAACCTAGAGAAAAGAAGATCTAGGTTTTCTGGCTCTTGATACCATGAAGGAACATTGATTGTATTGAAGTGTTAAGGGAATACATGGGAGATATATATAGGAGATATAGGAAGGAGTACTAATCCTAATAGGACTAGGATTAGTACACAGTAAATACTAATATTATTTTATAGTATTTATTTAATACTATCTGTTATTAAGGTCAAACTTTATTAATTATCTGAATTGCTATATTTGAAAGAAAGGAAGAATAAAAAAGAAAAATCACAATCCACACATAGTGATCATCAAAAAGAACCTCACCTTCCCCTCAAGTGAAAATAAGGTATGTGTATAGAGAAAATAAAAGTGATGTTTTTAATACTACAAAATCTAAAGCTTCATCTTAGTAGGTCCCACAAAAGATTTTTTGCCACCATATGTTTTTCTATATTAAACATCCAATCCCTCACTTCCCTCATTCCCTTCAACCAATATTCTCCTTTACTCTTCTTTATTGTAATTGCTTCCATTTCTCACTACACTTTTCAGATACACAGCCTGCAAAAACTGTCACTAGGCAGTTAGTATAAAACTTTTTAAGGTAGTTTAAACTTTTGTGTACATGAAGCTAGAAAGGAGGTATTCAATTATTACATTAACTTATATATAACTAGTCGATTATCTAGTTGTAGTTAAAAGAGTTGTGTGTAAATAGGTAAA
